# Efficacy of bougie first approach for endotracheal intubation with video laryngoscopy during continuous chest compression: a randomized crossover manikin trial

**DOI:** 10.1186/s12871-024-02560-3

**Published:** 2024-05-21

**Authors:** Xiaohan Xu, Haobo Ma, Yuelun Zhang, Wei Liu, Boris Jung, Xu Li, Le Shen

**Affiliations:** 1 Department of Anesthesiology, Peking Union Medical College Hospital, Chinese Academy of Medical Sciences, Peking Union Medical College , Beijing, 100730 China; 2 Department of Anesthesia, Critical Care and Pain Medicine, Beth Israel Deaconess Medical Center, Boston, MA 02215 USA; 3 Center for Prevention and Early Intervention, National Infrastructures for Translational Medicine, Institute of Clinical Medicine, Peking Union Medical College Hospital, Chinese Academy of Medical Sciences, Peking Union Medical College, Beijing, 100730 China; 4 Intensive Care Unit, Lapeyronie Teaching Hospital, Montpellier University, Montpellier, France; 5 Division of Pulmonary and Critical Care, Beth Israel Deaconess Medical Center, Harvard Medical School, Boston, MA 02115 USA

**Keywords:** Bougie, Endotracheal intubation, Cardiopulmonary resuscitation, Continuous chest compression, First-attempt success, Video laryngoscopy, Anesthesia resident, Manikin

## Abstract

**Background:**

Endotracheal intubation is challenging during cardiopulmonary resuscitation, and video laryngoscopy has showed benefits for this procedure. The aim of this study was to compare the effectiveness of various intubation approaches, including the bougie first, preloaded bougie, endotracheal tube (ETT) with stylet, and ETT without stylet, on first-attempt success using video laryngoscopy during chest compression.

**Methods:**

This was a randomized crossover trial conducted in a general tertiary teaching hospital. We included anesthesia residents in postgraduate year one to three who passed the screening test. Each resident performed intubation with video laryngoscopy using the four approaches in a randomized sequence on an adult manikin during continuous chest compression. The primary outcome was the first-attempt success defined as starting ventilation within a one minute.

**Results:**

A total of 260 endotracheal intubations conducted by 65 residents were randomized and analyzed with 65 procedures in each group. First-attempt success occurred in 64 (98.5%), 57 (87.7%), 56 (86.2%), and 46 (70.8%) intubations in the bougie-first, preloaded bougie, ETT with stylet, and ETT without stylet approaches, respectively. The bougie-first approach had a significantly higher possibility of first-attempt success than the preloaded bougie approach [risk ratio (RR) 8.00, 95% confidence interval (CI) 1.03 to 62.16, *P =* 0.047], the ETT with stylet approach (RR 9.00, 95% CI 1.17 to 69.02, *P =* 0.035), and the ETT without stylet approach (RR 19.00, 95% CI 2.62 to 137.79, *P =* 0.004) in the generalized estimating equation logistic model accounting for clustering of intubations operated by the same resident. In addition, the bougie first approach did not result in prolonged intubation or increased self-reported difficulty among the study participants.

**Conclusions:**

The bougie first approach with video laryngoscopy had the highest possibility of first-attempt success during chest compression. These results helped inform the intubation approach during CPR. However, further studies in an actual clinical environment are warranted to validate these findings.

**Trial registration:**

Clinicaltrials.gov; identifier: NCT05689125; date: January 18, 2023.

## Background

Establishing a definite airway through endotracheal intubation (ETI) helps to optimize oxygenation and ventilation, and improves patient outcome during cardiopulmonary resuscitation (CPR) in critically ill patients [1]. However, ETI during CPR can be challenging, particularly during the continuous chest compression, a key component of effective CPR [2–4]. Delayed intubation or intubation failure can lead to serious consequences [5]. Therefore, it is of utmost importance to identify an ETI approach with the highest likelihood of success during chest compression.

Video laryngoscopy has showed benefits for ETI during CPR compared to direct laryngoscopy [6–8]. However, the comparative effectiveness of different ETI approaches with video laryngoscopy, including the bougie first, preloaded bougie, endotracheal tube (ETT) with stylet, and ETT without stylet, needs to be further compared. Clinical trials have found that the bougie first approach had advantages over the ETT with stylet approach [9–11], and the ETT with stylet approach was superior to the ETT without stylet approach in non-CPR patients [12]. There was no significant difference between the preloaded bougie and the bougie first approaches in cadaveric models [13]. However, the effectiveness amongst the combinations of these approaches with video laryngoscopy has not been investigated in CPR patients or simulated chest compression scenarios.

Given the practical limitations of conducting clinical trial in CPR patients, we opted to conduct a randomized crossover manikin trial to assess the comparative effectiveness of the above four ETI approaches on first-attempt success with video laryngoscopy by anesthesia residents during simulated chest compression.

## Methods

### Study design

The study was a single-center, prospective, randomized, open-label, crossover simulation trial performed from February 1, 2023 to February 10, 2023 in Peking Union Medical College Hospital, a tertiary general teaching hospital in Beijing, China. The study was approved by the institutional review board of Peking Union Medical College Hospital, Beijing, China (reference number: K2562) on 10/11/2022 and registered in clinicaltrials.gov (identifier: NCT05689125) on 18/01/2023. Written informed consent was obtained from each participant. This article adheres to Consolidated Standards of Reporting Trials (CONSORT) guidelines.

### Study participants

We enrolled anesthesia residents in postgraduate year one to three in the Department of Anesthesiology, Peking Union Medical College Hospital in February 2023. Our residents had accumulated a minimum of five months of experience in endotracheal intubation, comprising approximately 200 intubations with an average of two intubations per day, prior to the commencement of the study. We excluded the residents who refused to participate and who failed the screening test (as detailed below). A research assistant recruited the residents and obtained informed consent.

### Screening test

We conducted a screening test and excluded the ones who failed the screening test to minimize the difference in residents’ competence of different ETI approaches. The residents performed ETI using bougie first, preloaded bougie, ETT with stylet, and ETT without stylet approaches with video laryngoscopy on a manikin. An attending anesthesiologist assisted them and assessed their performance. The device, intubation approaches, and performance assessment in the screening test were identical to those employed in the formal test (as detailed in “interventions” and “outcomes” below). The sole difference lay in the absence of chest compressions on the manikin during the screening test. A failure of the screening test was defined as being unable to ventilate the manikin within one minute after the insertion of a video laryngoscope blade into the mouth using any one of the four approaches.

### Randomization and allocation concealment

Each of the residents who passed the screening test performed ETIs using the four approaches in a randomized sequence generated by an epidemiologist without knowledge of participants inclusion or allocation using R (version 4.2.1, R Foundation for Statistical Computing, Vienna, Austria, 2022). After the resident passed the screening test and signed the informed consent, he/she was informed of the procedure sequence based on the predetermined random sequence.

### Interventions

ETIs were performed in a simulated chest compression scenario on a full-size adult manikin (“Airway Larry” with CPR Metrix & iPad®, CPR Savers & First Aid Supply, Arizona, USA) positioned supine on an operating table. Two research assistants alternately performed continuous chest compressions at a rate of 100 to 120 compressions per minute and a depth of 5 to 6 cm, which were continuously monitored by the sensor kit of the manikin. There is no interruption of chest compressions during ETI. The video laryngoscope (UEscope®, Zhejiang, China) used was featured with an angle-adjustable monitor and a blade angled at 40°. ETT (inner diameter 7.0 mm, Covidien Shiley™, Colorado, USA) cuffs were lubricated before intubation. An assistant provided necessary assistance during intubation. An attending anesthesiologist observed the performance without providing instructions. The residents had a five-minute break between each ETI. All the ETIs were recorded by a camera.

**Fig. 1:**
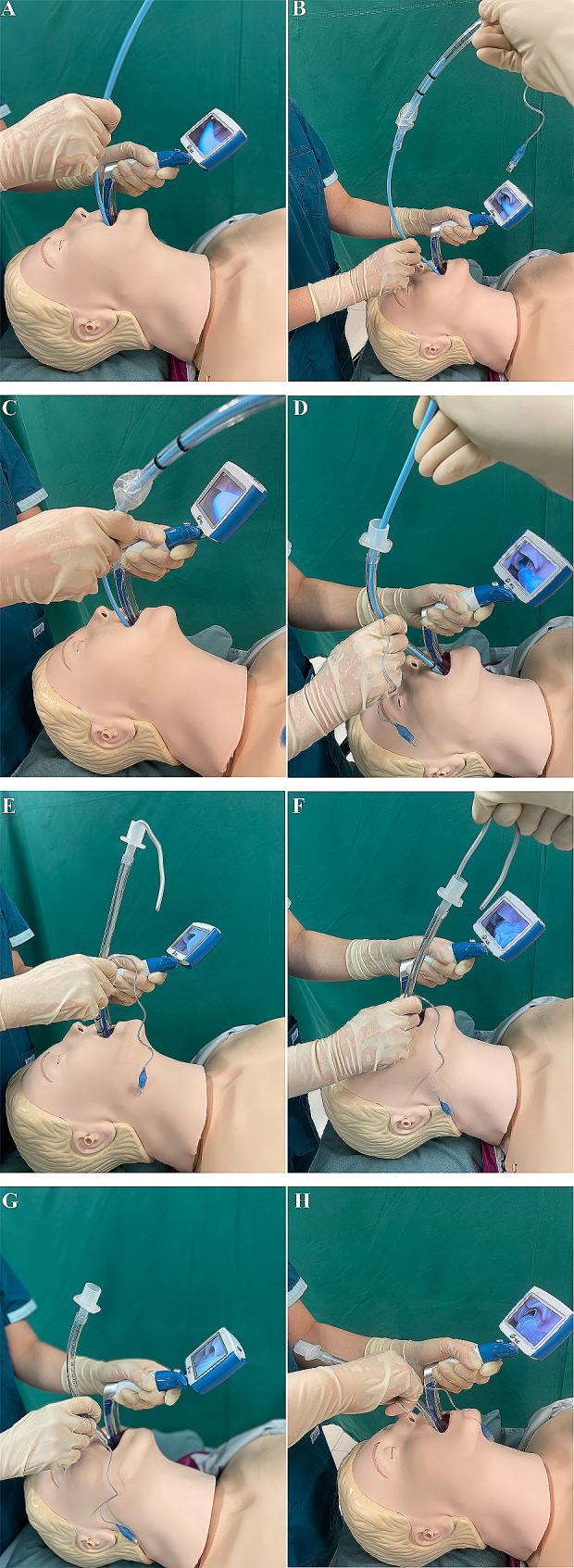
Endotracheal intubation approaches. **A** and **B**, bougie first; **C** and **D**, preloaded bougie; **E** and **F**, endotracheal tube with stylet; G and H, endotracheal tube without stylet

In the bougie first approach, the operator placed a bougie (15 Fr, SunMed®, Michigan, USA) into the trachea to a depth of about 25 cm (Fig. 1A). An assistant threaded an ETT over the bougie and helped secure the free end (Fig. 1B). While maintaining glottic visualization, the operator advanced the ETT over the bougie into the trachea and the assistant withdrew the bougie. In the preloaded bougie approach, the ETT was loaded onto a bougie prior to laryngoscopy. The operator placed the bougie into the trachea while holding the ETT in the intubating hand (Fig. 1C) to a depth of about 25 cm. Advancement of the ETT and withdrawal of the bougie were the same as that in the bougie first approach (Fig. 1D). In the ETT with stylet approach, a stylet (10 Fr, TuoRen™, Henan, China) was inserted into the ETT to shape a “hockey stick” bend at the cuff of 30° to 40°. Once the operator placed the ETT tip through the vocal cord, an assistant withdrew the stylet, while the operator continued to advance the ETT to an appropriate depth (Fig. 1E and F). In the ETT without stylet approach, the operator performed ETI without a bougie or a stylet in ETT (Fig. 1G and H).

### Outcomes

The primary outcome was success on the first attempt as a binary variable, which was defined as ventilating the manikin using an Ambu bag within one minute after the insertion of a video laryngoscope blade into the mouth with a single insertion of a bougie or an ETT into the mouth. An ETI attempt was terminated and defined as a failure when a resident tried for one minute but did not start ventilation, or when a resident felt unable to succeed and decided to give up in advance, or when a laryngoscope blade, a bougie, or an ETT was withdrawn out of the mouth after the initial insertion. The one-minute time restraint was added to represent the time pressure during CPR. If the first attempt of an approach failed, the resident would be given a second opportunity of the same approach. The outcome (success or failure) and duration of ETIs were measured based on the recorded videos of ETI procedures.

The secondary outcomes were success on two attempts, duration of the first attempt, overall intubation duration, and self-reported intubation difficulty. Success on two attempts included success on the first attempt and success on the second attempt after the failure of the first attempt. Duration of the first attempt was defined as the time elapsed between the insertion of a laryngoscope blade into the mouth and either the start of ventilation in a successful attempt or the termination of a failed attempt. Overall intubation duration was the duration of the first attempt if it succeeded, or the sum of the duration of both attempts if the first attempt failed. Self-reported intubation difficulty was a 0–10 ranking scale with 0 representing “the easiest” and 10 representing “the most difficult”.

### Statistical analysis

We determined the difference of the primary outcome in each comparison using a generalized estimating equation (GEE) logistic model to account for clustering of intubations operated by the same resident. Success on the first attempt was regressed against group allocation with log linkage. The autoregressive 1 correlation structure was selected since it had the smallest quasi-likelihood information criterion indicating the best fitness. Since each resident performed all the four ETI approaches, resident characteristics (such as postgraduate year) distributed equally among different approaches and thus did not cause confounding effects. To control the type I error in the multiple comparisons among four intervention groups, a hierarchical testing strategy was predefined before accessing the data. We first compared the bougie first group and the ETT without stylet group. If a significant difference was found, we next compared the bougie first group and the ETT with stylet group. If the primary outcome differed significantly between these two groups, we finally compared the bougie first group and the preloaded bougie group. Any non-significant results in the procedures would prevent the following tests and all the results in the following comparisons were regarded as insignificant. Risk ratio (RR) estimated from the GEE logistic model was reported as effect size measure, and the hierarchical testing was conducted based on the statistical tests for the dummy categorical intervention variables from the model at a two-sided α of 0.05.

For the secondary outcomes, failure on two attempts was analyzed using a GEE logistic model. Duration of the first attempt and overall intubation duration were analyzed using a Cox regression model in which a successful intubation was defined as an event and a cluster term of residents was included to calculate robust standard errors. A hazard ratio (HR) larger than 1 indicated shorter time to achieve the successful intubation. We used a mixed-effects linear regression model to compare the self-reported intubation difficulty between groups. Model fit was checked by the Q-Q plot of residuals from the model. If the Q-Q plot indicated potential skewed distribution, the outcome variable would be transformed using log function. No multiple comparison adjustment was conducted in the analyses of secondary outcomes; hence, these findings were regarded as exploratory results.

The sample size was calculated based on the results of a pilot study that included ten residents who had completed two-month rotations in the Department of Anesthesiology in the recent six months. Among the total 40 intubations in the pilot study, success on the first attempt occurred in 9 (90%), 7 (70%), 7 (70%), and 6 (60%) intubations in the bougie first, preloaded bougie, ETT with stylet, and ETT without stylet groups, respectively, indicating the smallest absolute differences between the bougie first group and any other group was 20% (90% vs. 70%). A sample of 260 intubations (65 residents) was deemed necessary to provide 80% power to detect an absolute difference of 20% in success on the first attempt with a 2-sided α of 0.05.

The statistical analysis was carried out using R (version 4.2.1, R Foundation for Statistical Computing, Vienna, Austria, 2022) along with the packages “pwr”, “geepack”, “survival”, and “lme4”.

## Results

**Fig. 2:**
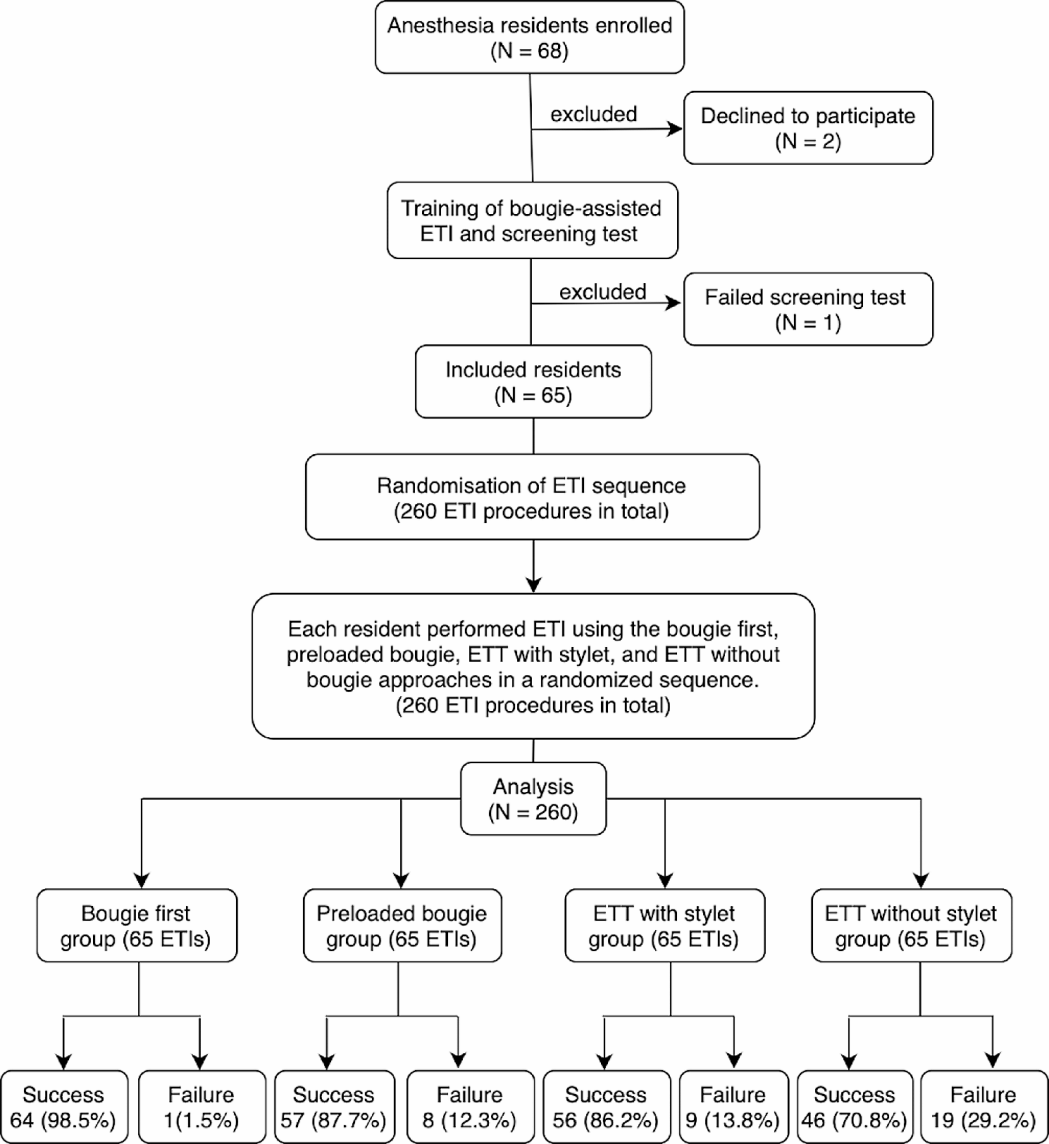
Flowchart of participant inclusion and summary of the primary outcome. Abbreviations: ETT, endotracheal tube; ETI, endotracheal intubation

A total of 68 eligible residents was recruited, but two residents declined to participate, and one resident was excluded in the screening test. Therefore, 65 residents were included in the trial and randomized. Each resident performed ETI procedures using the four approaches in randomized sequences, resulting in a total of 260 ETI procedures performed and analyzed in the trial with 65 in each group. The characteristics of the residents were presented in Table 1, and the flowchart of recruitment and randomization was shown in Fig. 2.

**Table 1:** Characteristics of randomized residents (*N* = 65)

Characteristics	Description
Grade	
PGY1	22 (33.8%)
PGY2	21 (32.3%)
PGY3	22 (33.8%)
Sex	
Male	17 (26.2%)
Female	48 (73.8%)
Age (year)	26 ± 2
Experience of ETI (time)	296 [92, 377]

Abbreviation: PGY, postgraduate year; ETI, endotracheal tube intubation. The categorial variable was described as number (percentage), normally distributed continuous variables were described as mean ± standardized deviation, and non-normally distributed continuous variables were described as median [interquartile range]

Success on the first attempt occurred in 64 (98.5%), 57 (87.7%), 56 (86.2%), and 46 (70.8%) intubations in the bougie first, preloaded bougie, ETT with stylet, and ETT without stylet groups, respectively (Fig. 2). The bougie first group had a significantly higher possibility of success on the first attempt than the ETT without stylet group [RR 19.00, 95% confidence interval (CI) 2.62 to 137.79, *P* = 0.004]. Next, in comparison with the ETT with stylet group, the possibility of success on the first attempt was significantly higher in the bougie first group (RR 9.00, 95% CI 1.17 to 69.02, *P =* 0.035). Finally, the bougie first group also had a higher possibility of success on the first attempt when compared with the preloaded bougie group (RR 8.00, 95% CI 1.03 to 62.16, *P =* 0.047) (Table 2).

**Table 2:** Comparison of the primary outcome (*N* = 260)

Comparison	RR		*P*
	Estimate	95% CI	
Bougie first vs. Preloaded bougie	8.00	1.03 to 62.16	0.047^*^
Bougie first vs. ETT with stylet	9.00	1.17 to 69.02	0.035^*^
Bougie first vs. ETT without stylet	19.00	2.62 to 137.79	0.004^*^

Abbreviations: vs., versus; RR, risk ratio; CI, confidence interval; ETT, endotracheal tube; ^*^P < 0.05. The primary outcome was analyzed by the generalized estimating equation logistic model

The secondary outcomes of the four groups were described in Table 3. The possibility of success on two attempts was higher in the bougie first group than in the ETT without stylet group in the GEE logistic model (98.5% vs. 84.6%, RR 10.00, 95% CI 1.28 to 78.12, *P* = 0.028, Tables 3 and 4). Duration of the first attempt was significantly shorter in the bougie first group than in the preloaded bougie group (21 s vs. 26 s, HR 1.62, 95% CI 1.28 to 2. 04, *P* < 0.001) or in the ETT without stylet group (21 s vs. 27 s, HR 2.09, 95% CI 1.51 to 2.89, *P* < 0.001) in the Cox regression model (Tables 3 and 4). Similarly, the bougie first group had a shorter overall intubation duration than the preloaded bougie group (21 s vs. 26 s, HR 1.61, 95% CI 1.28 to 2.01, *P* < 0.001) or the ETT without stylet group (21 s vs. 27 s, HR 2.12, 95% CI 1.54 to 2.91, *P* < 0.001) (Tables 3 and 4). Residents reported that the bougie first approach was the easier than the preloaded bougie approach (0–10 scale of difficulty: 3 vs. 4, mean difference − 0.86, 95% CI -1.57 to -0.15, *P* = 0.018) and the ETT without stylet approach (3 vs. 7, mean difference − 3.18, 95% CI -3.89 to -2.48, *P* < 0.001) in the mixed-effects linear model (Tables 3 and 4).

**Table 3:** Description of the secondary outcomes (*N* = 260)

Outcome/ Group	Bougie first	Preloaded bougie	ETT with stylet	ETT without stylet
Success on two attempts	64 (98.5%)	62 (95.4%)	63 (96.9%)	55 (84.6%)
Duration of the first attempt (s)	21 [16, 27]	26 [19, 37]	16 [13, 26]	27 [16, 47]
Overall intubation duration (s)	21 [16, 27]	26 [19, 37]	17 [14, 27]	28 [17, 47]
Self-reported intubation difficulty	3 [1, 5]	4 [2, 6]	3 [2, 4]	7 [4, 9]

Abbreviation: ETT, endotracheal tube. The categorial variable was described as number (percentage), and non-normally distributed continuous variables were described as median [interquartile range]

**Table 4:** Comparisons of the secondary outcomes (*N* = 260)

Group	Estimate^a^	95% CI	*P*
Success on two attempts			
Bougie first vs. Preloaded bougie	3.00	0.31 to 28.84	0.341
Bougie first vs. ETT with stylet	2.00	0.18 to 22.06	0.571
Bougie first vs. ETT without stylet	10.00	1.28 to 78.12	0.028^*^
Duration of the first attempt (s)			
Bougie first vs. Preloaded bougie	1.62	1.28 to 2. 04	< 0.001^*^
Bougie first vs. ETT with stylet	0.96	0.64 to 1.44	0.832
Bougie first vs. ETT without stylet	2.09	1.51 to 2.89	< 0.001^*^
Overall intubation duration (s)			
Bougie first vs. Preloaded bougie	1.61	1.28 to 2.01	< 0.001^*^
Bougie first vs. ETT with stylet	1.04	0.68 to 1.58	0.864
Bougie first vs. ETT without stylet	2.12	1.54 to 2.91	< 0.001^*^
Self-reported intubation difficulty			
Bougie first vs. Preloaded bougie	-0.86	-1.57 to -0.15	0.018^*^
Bougie first vs. ETT with stylet	-0.02	-0.72 to 0.69	0.966
Bougie first vs. ETT without stylet	-3.18	-3.89 to -2.48	< 0.001^*^

Abbreviations: CI, confidence interval; ETT, endotracheal tube. ^a^Success on two attempts was analyzed by a generalized estimating equation logistic model. Duration of the first attempt and overall intubation duration were analyzed by Cox regression model including a cluster term of residents. Self-reported intubation difficulty was analyzed by a linear mixed-effects model. The estimates were risk ratio for logistic model, hazard ratio for Cox model, and mean difference for linear model. ^*^P < 0.005

## Discussion

In this randomized crossover trial, we found that during continuous chest compression, the bougie first approach with video laryngoscopy demonstrated a significantly higher likelihood of first-attempt intubation success compared to the preloaded bougie, ETT with stylet, and ETT without stylet approaches on the manikin. Importantly, the bougie first approach did not result in prolonged intubation or increased self-reported difficulty in the study participants.

The Guidelines of the American Society of Anesthesiologists and the Difficult Airway Society did not specify on the ETI approaches during CPR [14, 15]. Previous simulation trials have illustrated the effectiveness of bougie-assisted ETI via direct laryngoscopy on the adult and infant manikins in CPR scenarios [16–18]. Our trial identified the most effective intubation approach on a manikin during continuous chest compression, providing simulation evidence to support the choice of bougie-first approach with video laryngoscopy for ETI during CPR, a more chaotic situation.

The major benefits of bougie for intubation is its smaller diameter and malleable construction. The bougie has a diameter of 5 mm, while the outer diameter of ETT (ID 7.0 mm) is about 9.3 mm, even without considering the cuff size. The smaller profile of bougie reduces the obstruction of the view of the glottic opening, and the malleable shaft makes it easier to handle the higher Cormack-Lehane grade view [9, 15]. This advantage is particularly important when using the video laryngoscopy, as its blade is bulkier comparing to the direct laryngoscope, and leaves a more restricted space in oropharynx. Furthermore, in spite of the improved glottic view under video laryngoscopy, dedicated expertise is required to direct and advance the ETT into the trachea in the camera view, possibly due to the even more exacerbated misalignment between oral-pharyngeal-laryngeal axis and direct vision line [19]. Those reasons may explain why the video laryngoscopy did not show advantages in several clinical trials when the bougie was not used to facilitate ETI [20–23].

In addition, the bougie can be bent prior to intubation and stay in that configuration, while the ETT requires a stylet to help to stay in a “hockey stick” shape. This explains why the ETT without stylet approach had the lowest first-attempt success. The bougie can be placed into the trachea and serve as a guide for the passage of the ETT, while the stylet needs to be withdrawn after the ETT tip reaches the glottic inlet. Therefore, it is easier to advance the ETT through the moving glottic inlet over a bougie as a guide during continuous chest compression, which was confirmed by our results that the bougie first approach had higher first-pass success than the ETT with stylet approach.

Our study also indicated the bougie first approach had advantages over the preloaded bougie approach for ETI during chest compression. While the preloaded bougie approach can save time by eliminating the need to load an ETT during intubation, the preloaded ETT adds weight on the distal end of the bougie and can increase the challenge in directing the bougie tip [13]. This may explain why the bougie first approach had higher first-attempt success than the preloaded bougie approach.

Our study was limited in the following aspects. First, ETI on the manikin is different from that on real patients due to the fidelity limitations of the manikin. The manikin utilized in our study was unable to fully replicate airway secretions found in real patients, nor could it detect bronchial intubation resulting from incorrect intubation depth. The ETI during real CPR would be much more challenging. It is thus important to establish the comparative effectiveness of different approaches during simulated chest compression at the first stage. That was also the reason why we added a one-minute time limitation to deem a successful ETI, which created time urgency in the simulation test. We believed the edge conferred by the bougie-first approach in a simulated scenario could be magnified in a real and chaotic CPR situation. Second, we acknowledged the potential for learning effects due to the crossover design. We did not incorporate a washout period as the residents had previously utilized the same manikin and video laryngoscope during the screening test, which resulted in their prior familiarity with the platform. The randomized intubation sequence further minimized learning effects. Third, despite the sufficient statistical power, the absolute differences of first-attempt success between groups were not very large, possibly because we only included residents who passed the screening test. This also implied that adequate training is a necessary component for successful intubation in precarious situations. Forth, our participants, although residents, had amassed experience with a minimum of 200 ETIs by the time of this study. Nonetheless, caution should be employed when generalizing our findings to physicians of varying proficiency levels. Finally, we used UEscope, which has an upward blade angle similar to the Macintosh laryngoscope but less than the McGrath or Glidescope [24]. However, we believe similar results could be replicated if the users are familiar with any particular video laryngoscopes.

## Conclusions

This study showed the bougie first approach with video laryngoscopy provides the higher first-pass success on a manikin during simulated chest compression. Further clinical study is necessary to validate this conclusion in CPRs.

## Data Availability

The datasets used and/or analyzed during the current study and the full trial protocol are available from the corresponding author on reasonable request.

## Abbreviations

ETI: Endotracheal intubation
CPR: Cardiopulmonary resuscitation
ETT: Endotracheal tube
GEE: Generalized estimating equation
RR: Risk ratio
CI: Confidence interval
HR: Hazard ratio
